# Publisher Correction: Chromatin dysregulation and DNA methylation at transcription start sites associated with transcriptional repression in cancers

**DOI:** 10.1038/s41467-019-10557-7

**Published:** 2019-05-29

**Authors:** Mizuo Ando, Yuki Saito, Guorong Xu, Nam Q. Bui, Kate Medetgul-Ernar, Minya Pu, Kathleen Fisch, Shuling Ren, Akihiro Sakai, Takahito Fukusumi, Chao Liu, Sunny Haft, John Pang, Adam Mark, Daria A. Gaykalova, Theresa Guo, Alexander V. Favorov, Srinivasan Yegnasubramanian, Elana J. Fertig, Patrick Ha, Pablo Tamayo, Tatsuya Yamasoba, Trey Ideker, Karen Messer, Joseph A. Califano

**Affiliations:** 1 Moores Cancer Center, University of California San Diego, 3855 Health Sciences Dr, La Jolla, CA 92093 USA; 2 Department of Otolaryngology - Head and Neck Surgery, Graduate School of Medicine, University of Tokyo, 7-3-1 Hongo, Bunkyo-ku, Tokyo 113-8655 Japan; 3 Department of Medicine, Center for Computational Biology and Bioinformatics, University of California San Diego, 9500 Gilman Drive, La Jolla, CA 92093 USA; 4 Department of Medicine, University of California San Diego, 9500 Gilman Drive, La Jolla, CA 92093 USA; 5 Department of Medicine (Oncology), Stanford University School of Medicine, 875 Blake Wilbur Dr, Palo Alto, CA 94304 USA; 6 Department of Otolaryngology - Head and Neck Surgery, Johns Hopkins University School of Medicine, 601 N Caroline St, Baltimore, MD 21287 USA; 7 Department of Oncology, Sidney Kimmel Comprehensive Cancer Center, Johns Hopkins University School of Medicine, 401 N Broadway, Baltimore, MD 21231 USA; 8 Laboratory of Systems Biology and Computational Genetics, Vavilov Institute of General Genetics, Russian Academy of Sciences, Gubkina str. 3, Moscow, 119333 Russia; 9 Department of Otolaryngology - Head and Neck Surgery, University of California San Francisco, 2380 Sutter St, San Francisco, CA 94115 USA; 10 Division of Otolaryngology - Head and Neck Surgery, Department of Surgery, University of California San Diego, 9300 Campus Point Drive, La Jolla, CA 92037 USA

**Keywords:** Breast cancer, Cancer epigenetics, Colon cancer, Head and neck cancer

Correction to: *Nature Communications* 10.1038/s41467-019-09937-w, published online 16 May 2019.

The original version of this Article contained an error in the author affiliations.

Trey Ideker was incorrectly associated with ‘Department of Medicine (Oncology), Stanford University School of Medicine, 875 Blake Wilbur Dr, Palo Alto, CA 94304, USA.’

This has now been corrected in both the PDF and HTML versions of the Article.

