# Supplementary material for: Chromatin dysregulation and DNA methylation at transcription start sites associated with transcriptional repression in cancers
Source: Nat Commun. 2019 May 16;10:2188. doi: 10.1038/s41467-019-09937-w (PMC6522544; doi:10.1038/s41467-019-09937-w)
Supplement: Supplementary file 1 — Supplementary Information [file 41467_2019_9937_MOESM1_ESM.pdf]

Supplementary Information

Chromatin dysregulation and DNA methylation at transcription start sites associated with transcriptional repression in cancers

Ando et al.

Supplementary Figure 1.

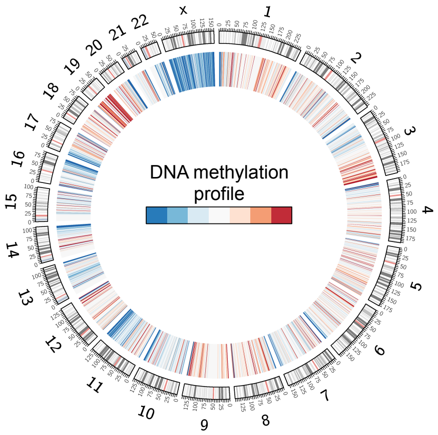

Supplementary Figure 1. Circos plot of genome-wide DNA methylation changes. The red (high in HPVOPSCC) and blue (high in normal oropharynx) indicate the DNA methylation difference in 1Mbp windows. The outer track is a chromosome cytogenetic band ideogram with centromeres shaded in red.

Supplementary Figure 2.

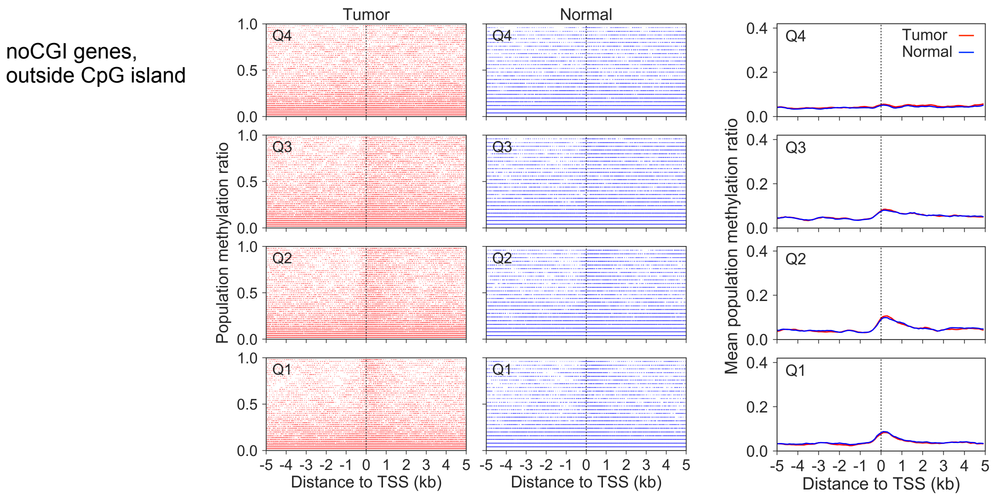

Supplementary Figure 2. DNA methylation and gene expression in HPVOPSCC and normal oropharynx. Scatter plots separated by gene expression quartiles based on the expression levels in either tumor or normal samples (n= 1677, 1677, 1677, 1677 for Q1, Q2, Q3, Q4, respectively; Q4 is the highest expression) showing methylation ratio at 100 bp segments including genomic loci outside CpG islands in noCGI genes (n=6708, left panels). Right panels show the mean values. TSS, transcription start site.

Supplementary Figure 3.

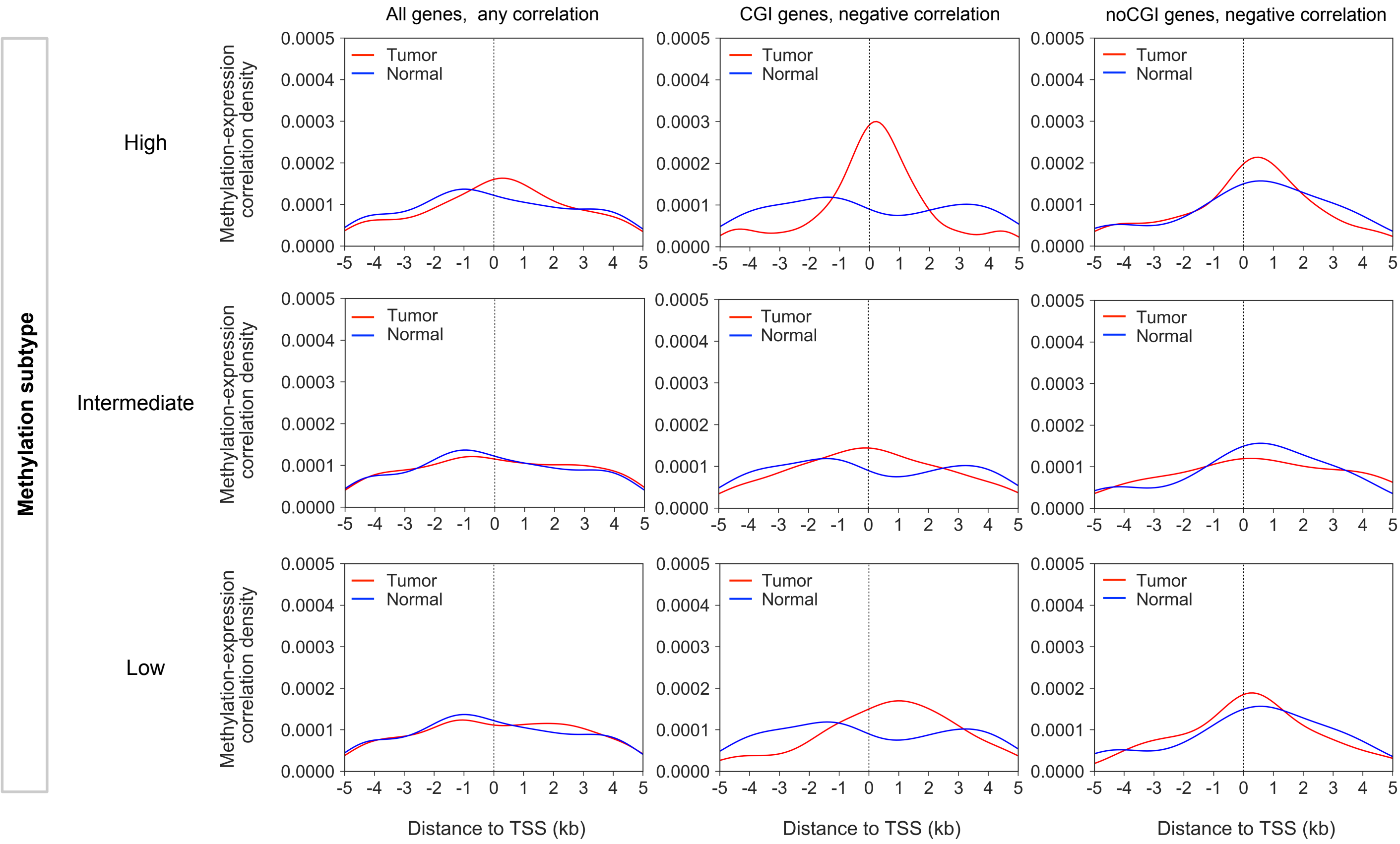

Supplementary Figure 3. DNA methylation-expression correlation density plots of high, intermediate, and low methylation subtypes separately. Y axis represents probability and the area under the curve adds up to 1. Plots of any correlation in all genes (n=19866) in our discovery HPVOPSCC dataset, and negative correlation in CGI genes (n=13158) and noCGI genes (n=6708) are shown.

Supplementary Figure 4.

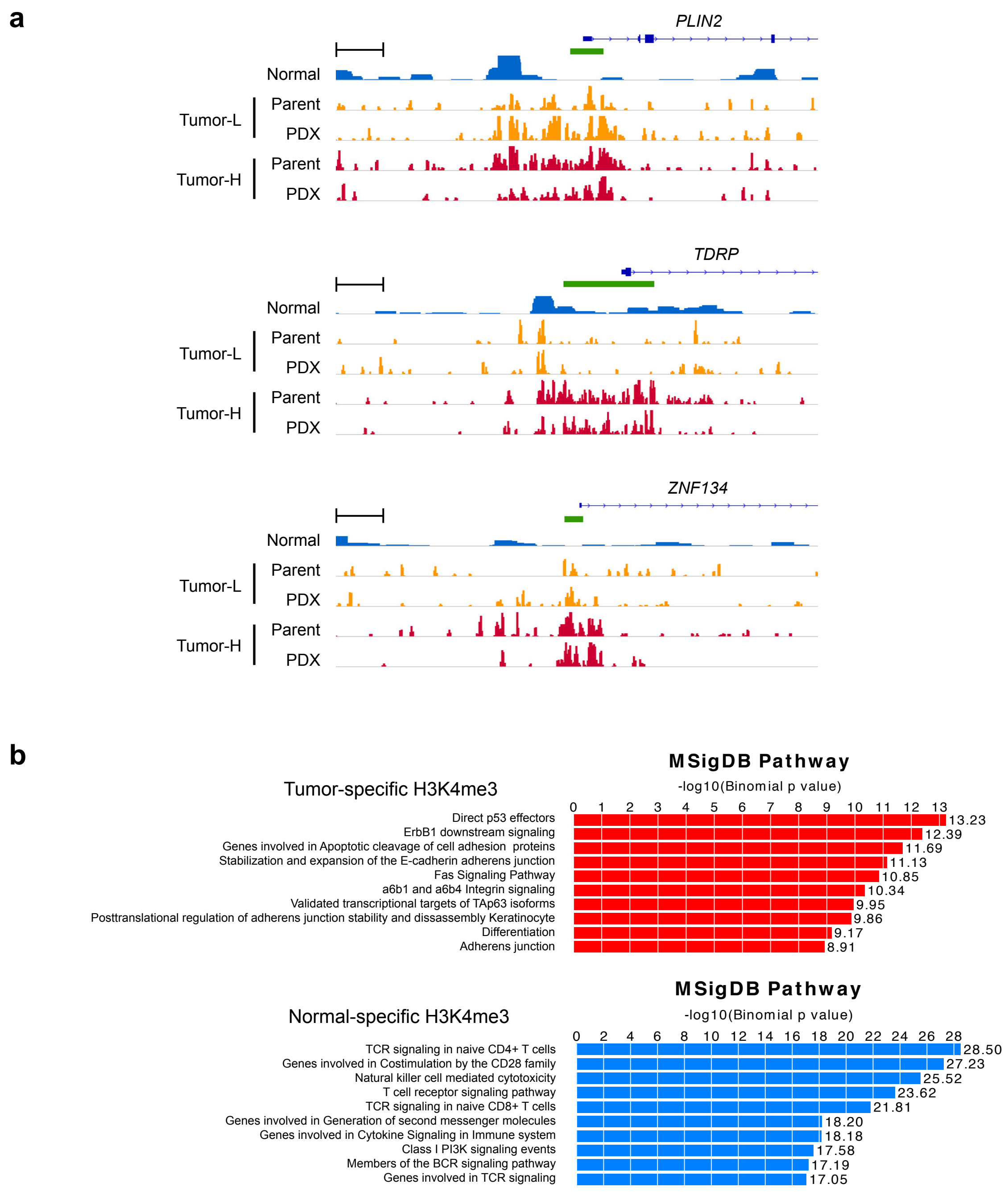

Supplementary Figure 4. Characteristics of PDXs. **a**, MBD-seq data of normal tissue, PDXs, and the parental tumors for three representative genes in Fig. 3a. Note the similar methylation profiles of these first passage (F1) PDXs to the tumors from which they were derived. Genes (blue) and CpG islands (green) are presented in a forward fashion. Scale bars, 1kb. **b**, Top 10 significantly enriched pathways among tumor (PDXs)- and normal-specific H3K4me3 peaks using GREAT algorithm.

Supplementary Figure 5.

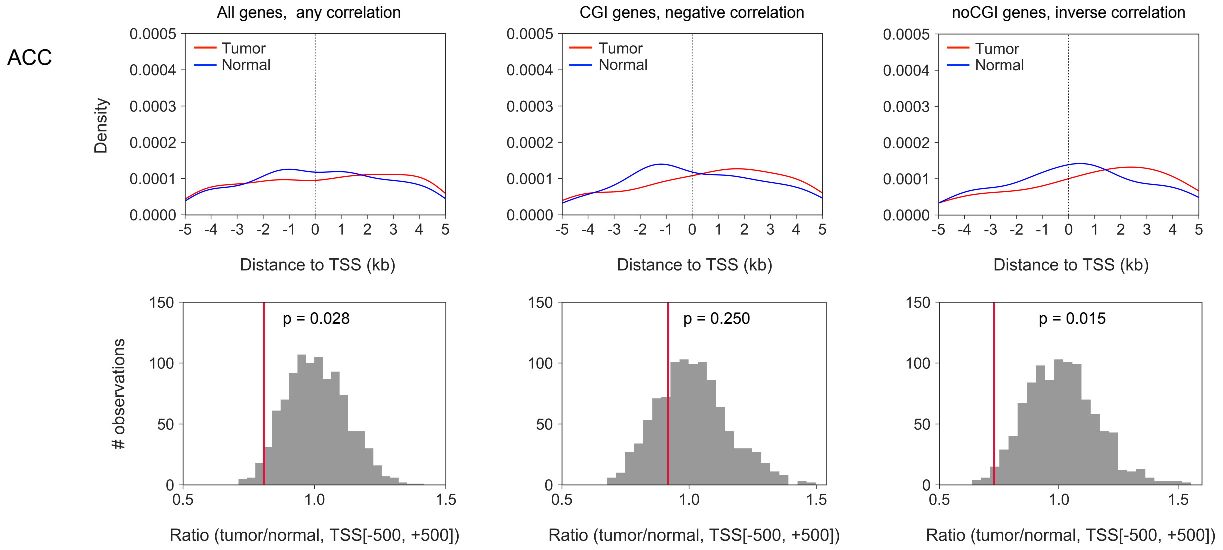

Supplementary Figure 5. Association of DNA methylation with gene expression in primary salivary gland adenoid cystic carcinoma (ACC) and normal parotid samples. Analogous MBD-seq and RNA-seq datasets in our discovery cohort were used. Plots of any correlation in all genes (n=19239) in the ACC dataset, and negative correlation in CGI genes (n=12628) and noCGI genes (n=6611) are shown.

Supplementary Figure 6.

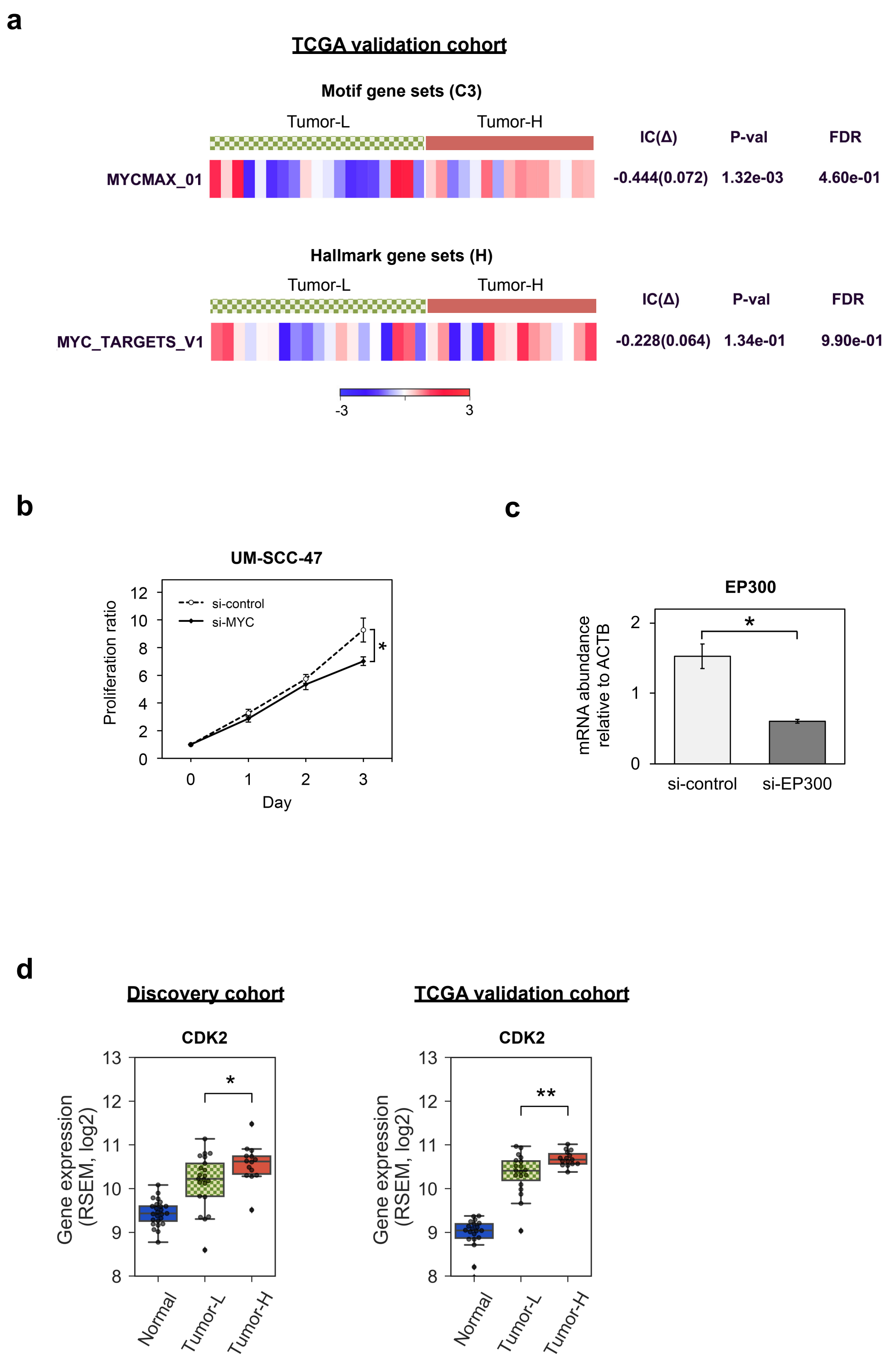

Supplementary Figure 6. (cont.)

e

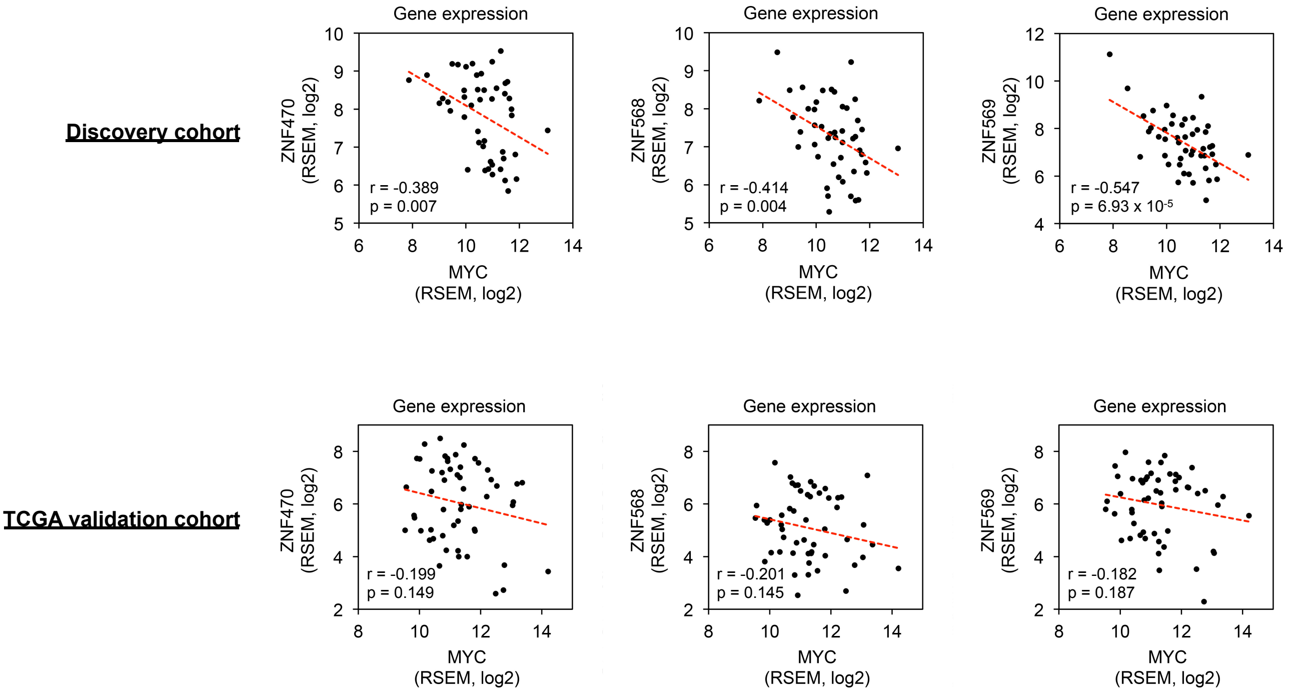

Supplementary Figure 6. Deregulation of MYC pathway associated with highly methylated HPVOPSCC subtype. **a**, Single-sample gene set enrichment analysis (ssGSEA) scores ranked by their degree of association (IC) between highly and lowly methylated HPVOPSCC tumors. MYC-related motif (C3) and hallmark (H) gene sets enriched in highly methylated tumors in the TCGA validation cohort. An empirical phenotype-based permutation test procedure is used to estimate P values. Tumor-H, highly methylated HPVOPSCC. Tumor-L, lowly methylated HPVOPSCC. **b**, UM-SCC-47 cell viability assay measured after *MYC* knockdown. Growth is normalized to day 0 and measured over 3 days. Experiments were performed six times, and data are expressed as mean ± SE. * P < 0.05, t-test. **c**, Confirmation of *EP300* knockdown with siRNA. mRNA and protein detection were performed on lysates of UM-SCC-47 cells collected at 48 hours after siRNA transfection. Experiments were performed in triplicate, and data are expressed as mean ± SE. * P < 0.05, t-test. **d**, CDK2 expression of each sample in our discovery cohort (n=35, excluding normal samples) and the TCGA validation cohort (n=34, excluding normal samples). * P < 0.05 and ** P < 0.01, t-test. In boxplots, the ends of the boxes and the middle line represent the lower and upper quartiles, and medians, respectively. Whiskers extend to show the rest of the distribution, except for points that are determined to be outliers using a function of the inter-quartile range. **e**, Scatter plots of three representative genes whose expression level was negatively correlated with MYC expression both in our discovery and the TCGA validation cohorts. Pearson's correlation coefficient. Source data are provided as a Source Data file.

Supplementary Table 1. Primers used for ChIP-qPCR in Figure 6e.

Location		Sequence
-2.2kb	Forward	ATGTTCATTAGCAGTGGTGATAGGTTA
	Reverse	AGGTGACTATTCAACCGCATAAGAG
-1.0kb	Forward	TCTCCCGTCTAGCACCTTTGA
	Reverse	TTGCAACAGTCTCGGGCTG
+0.1kb	Forward	GAGGGATCGCGCTGAGTATAAA
	Reverse	GCGAGTTAGATAAAGCCCCGA
+2.2kb	Forward	ATGCCCCTCAACGTTAGCTTC
	Reverse	ACCGAGTCGTAGTCGAGGTCAT

Supplementary Table 2. Primers used for ChIP-qPCR in Figure 6f.

Gene		Sequence
GCK	Forward	CACTGTTCGTGGGACTGTAAA
	Reverse	GATGGCTGGGTCAAATGGTA
CDK2	Forward	CCAAGAACCAAACTTGCCTGG
	Reverse	CCCCAGGCCTTTCTATTGGT
CDK4	Forward	GAGCGACCCTTCCATAACCA
	Reverse	GGGCTGGCGTGAGGTAAGT
NPM1	Forward	CTCGTGAGCCAGGGATGCT
	Reverse	CCCTAGTGCTACCAGCCTCTTAAC

Supplementary Table 3. TaqMan primers and probes used for qMSP in Figure 6g.

Gene		Sequence
ACTB	Forward	TGGTGATGGAGGAGGTTTAGTAAGT
	Reverse	AACCAATAAAACCTACTCCTCCCTTAA
	Probe	ACCACCACCCAACACACA
ZNF470	Forward	CGTTTGCGTGTATGGTTCGG
	Reverse	AACCTCCTCCGACCGAATTT
	Probe	TGCGGAAGGCGGTGTGTGTTGGAAT
ZNF568	Forward	GTTAGTTTTTCGTTTGGGTTTATTC
	Reverse	GAAAAACTCATCTCCATTACGC
	Probe	AGTTGCGGGTCGTCGTAGC
ZNF569	Forward	GTGCGAGTGGAGGTTGGTAT
	Reverse	TGTGATTGGGTGCGAGGAAG
	Probe	TAGTGCGAGGTTGTTATTTCGTGTGTTT
