# Supplementary material for: Chromatin dysregulation and DNA methylation at transcription start sites associated with transcriptional repression in cancers
Source: Nat Commun. 2019 May 16;10:2188. doi: 10.1038/s41467-019-09937-w (PMC6522544; doi:10.1038/s41467-019-09937-w)
Supplement: Supplementary file 3 — Description of Additional Supplementary Files [file 41467_2019_9937_MOESM3_ESM.pdf]

Description of Additional Supplementary Information

File Name: Supplementary Data 1
Description: List of selected 59 genes that exhibited strongly significant (FDR $q<0.001$) negative correlation between DNA methylation and RNA expression levels in the discovery cohort.

File Name: Supplementary Data 2
Description: Lists of genes leading to the enrichment of pathways in the discovery cohort: motif gene sets (C3).

File Name: Supplementary Data 3
Description: Lists of genes leading to the enrichment of pathways in the discovery cohort: hallmark gene sets (H).

File Name: Supplementary Data 4
Description: Lists of genes leading to the enrichment of pathways in the discovery cohort: curated gene sets (C2).

File Name: Supplementary Data 5
Description: a) MuTect analysis. b) VarDict analysis.
