# Supplementary material for: Chromatin dysregulation and DNA methylation at transcription start sites associated with transcriptional repression in cancers
Source: Nat Commun. 2019 May 16;10:2188. doi: 10.1038/s41467-019-09937-w (PMC6522544; doi:10.1038/s41467-019-09937-w)
Supplement: Supplementary file 9 — Reporting Summary [file 41467_2019_9937_MOESM9_ESM.pdf]

nature research

Corresponding author(s): Joseph A. Califano

Reporting Summary

Nature Research wishes to improve the reproducibility of the work that we publish. This form provides structure for consistency and transparency in reporting. For further information on Nature Research policies, see Authors & Referees and the Editorial Policy Checklist.

Statistical parameters

When statistical analyses are reported, confirm that the following items are present in the relevant location (e.g. figure legend, table legend, main text, or Methods section).

n/a	Confirmed	
☐	☒	The exact sample size (*n*) for each experimental group/condition, given as a discrete number and unit of measurement
☐	☒	An indication of whether measurements were taken from distinct samples or whether the same sample was measured repeatedly
☐	☒	The statistical test(s) used AND whether they are one- or two-sided<br>*Only common tests should be described solely by name; describe more complex techniques in the Methods section.*
☒	☐	A description of all covariates tested
☐	☒	A description of any assumptions or corrections, such as tests of normality and adjustment for multiple comparisons
☐	☒	A full description of the statistics including central tendency (e.g. means) or other basic estimates (e.g. regression coefficient) AND variation (e.g. standard deviation) or associated estimates of uncertainty (e.g. confidence intervals)
☒	☐	For null hypothesis testing, the test statistic (e.g. *F*, *t*, *r*) with confidence intervals, effect sizes, degrees of freedom and *P* value noted<br>*Give P values as exact values whenever suitable.*
☒	☐	For Bayesian analysis, information on the choice of priors and Markov chain Monte Carlo settings
☒	☐	For hierarchical and complex designs, identification of the appropriate level for tests and full reporting of outcomes
☒	☐	Estimates of effect sizes (e.g. Cohen's *d*, Pearson's *r*), indicating how they were calculated
☐	☒	Clearly defined error bars<br>*State explicitly what error bars represent (e.g. SD, SE, CI)*

Our web collection on statistics for biologists may be useful.

Software and code

Policy information about availability of computer code

Data collection	No software was used other than those listed in the Methods section.
Data analysis	MapSplice2 version 2.0.1.9, RSEM version 1.2.9, MACS version 1.4.4, Circos version 0.6.9, HOMER v4.8, Bowtie v1.0.1, GREAT version 3.0.0, cfncluster v1.3.1, BWA-mem v.0.7.12, SAMtools v.1.1, Picard Tools v.1.96, GATK v2.4-9, MuTect version 1.1.5, VarDict 2017.11.23, ANNOVAR 2017Jun01, Integrative Genomics Viewer version 2.4, R version 3.2.1, Python 2.7.13 were used to analyze and plot the data.

For manuscripts utilizing custom algorithms or software that are central to the research but not yet described in published literature, software must be made available to editors/reviewers upon request. We strongly encourage code deposition in a community repository (e.g. GitHub). See the Nature Research guidelines for submitting code & software for further information.

Data

Policy information about availability of data

All manuscripts must include a data availability statement. This statement should provide the following information, where applicable:

- Accession codes, unique identifiers, or web links for publicly available datasets
- A list of figures that have associated raw data
- A description of any restrictions on data availability

The datasets generated from RNA-seq, MBD-seq, and ChIP-seq are available in the Gene Expression Omnibus repository with the following accession numbers:

GSE112026 (RNA-seq), GSE112023 (MBD-seq), and GSE112021 (ChIP-seq). All other remaining data supporting the findings of this study are available within the Article and Supplementary Files, or available from the authors upon reasonable request.

Field-specific reporting

Please select the best fit for your research. If you are not sure, read the appropriate sections before making your selection.

☒ Life sciences ☐ Behavioural & social sciences ☐ Ecological, evolutionary & environmental sciences

For a reference copy of the document with all sections, see nature.com/authors/policies/ReportingSummary-flat.pdf

Life sciences study design

All studies must disclose on these points even when the disclosure is negative.

Sample size	No statistical methods were used to determine sample size. Primary tumor and normal oropharynx tissue samples for the discovery analysis were obtained according to the previously described cohort from the Johns Hopkins Tissue Core, part of the Head and Neck Cancer Specialized Program of Research Excellence. Samples for validation analysis were collected as many as possible from the publicly available TCGA cohorts.
Data exclusions	In principle, we used samples with high-quality sequencing data from both MBD-seq and RNA-seq. One sample with a failed whole-exome sequencing due to insufficient DNA quality was excluded from the analysis.
Replication	All attempts at replication were successful.
Randomization	No randomization of participants. Primary tumor and normal oropharynx tissue samples for the discovery analysis were obtained according to the previously described cohort.
Blinding	The investigators were not blinded during data collection and analysis.

Reporting for specific materials, systems and methods

Materials & experimental systems

n/a	Involved in the study
☒	☐ Unique biological materials
☐	☒ Antibodies
☐	☒ Eukaryotic cell lines
☒	☐ Palaeontology
☐	☒ Animals and other organisms
☐	☒ Human research participants

Methods

n/a	Involved in the study
☐	☒ ChIP-seq
☒	☐ Flow cytometry
☒	☐ MRI-based neuroimaging

Antibodies

Antibodies used	c-Myc (D3N8F), GAPDH (14C10), H3K4me3 (C42D8), H3K9me3 (D4W1U), and H3K27ac (D5E4); Cell Signaling Technology. Cdk2 (M2, sc-163); Santa Cruz Biotechnology).
Validation	[c-Myc] https://www.cellsignal.com/products/primary-antibodies/c-myc-d3n8f-rabbit-mab/13987<br>[GAPDH] https://www.cellsignal.com/products/primary-antibodies/gapdh-14c10-rabbit-mab/2118<br>[H3K4me3] https://www.cellsignal.jp/products/primary-antibodies/tri-methyl-histone-h3-lys4-c42d8-rabbit-mab/9751<br>[H3K9me3] https://www.cellsignal.com/products/primary-antibodies/tri-methyl-histone-h3-lys9-d4w1u-rabbit-mab/13969<br>[H3K27ac] https://www.cellsignal.com/products/primary-antibodies/acetyl-histone-h3-lys27-d5e4-xp-rabbit-mab/8173<br>[Cdk2] https://www.scbt.com/scbt/product/cdk2-antibody-m2

Eukaryotic cell lines

Policy information about cell lines

Cell line source(s)	UM-SCC-47 was obtained from the Gutkind Laboratory at the University of California San Diego (Martin, D. et al. Oncotarget 2014;5:8906-8923).

Authentication	Cells have been authenticated by short tandem repeat (STR) profiling.
Mycoplasma contamination	Cells have been tested by PCR for mycoplasma contamination.
Commonly misidentified lines (See ICLAC register)	The study did not involve commonly misidentified lines.

Animals and other organisms

Policy information about studies involving animals; ARRIVE guidelines recommended for reporting animal research

Laboratory animals	Immunodeficient mice, BALB/c Nude
Wild animals	The study did not involve wild animals.
Field-collected samples	The study did not involve samples collected from the field.

Human research participants

Policy information about studies involving human research participants

Population characteristics	Primary tumor tissue samples were obtained from a cohort of 47 patients with HPV-related oropharyngeal squamous cell carcinoma, as previously described. For comparison, healthy oropharynx mucosal tissue from uvulopalatopharyngoplasty (UPPP) surgical specimens were obtained from 25 cancer-unaffected controls. (Guo T et al. Int J Cancer 2016;139:373-82)
Recruitment	All tissue samples were collected from the Johns Hopkins Tissue Core, part of the Head and Neck Cancer Specialized Program of Research Excellence (HNC-SPORE). All patients were recruited under an institutional review board approved protocol (#NA_00-36235). (Guo T et al. Int J Cancer 2016;139:373-82)

ChIP-seq

Data deposition

☒ Confirm that both raw and final processed data have been deposited in a public database such as GEO.

☒ Confirm that you have deposited or provided access to graph files (e.g. BED files) for the called peaks.

Data access links *May remain private before publication.*	https://www.ncbi.nlm.nih.gov/geo/query/acc.cgi?acc=gse112021
Files in database submission	GSM3510045, GSM3510046, GSM3510047, GSM3510048, GSM3510049, GSM3510050, GSM3510051, GSM3510052, GSM3510053, GSM3510054, GSM3510055, GSM3510056, GSM3510057, GSM3510058, GSM3510059, GSM3510060, GSM3510061, GSM3510062, GSM3510063, GSM3510064, GSM3510065, GSM3510066, GSM3510067, GSM3510068, GSM3510069, GSM3510070
Genome browser session (e.g. UCSC)	No longer applicable

Methodology

Replicates	Each sample was analyzed once.
Sequencing depth	ChIP-DNA was sequenced with a coverage of approximately 45,000,000 X and paired-end reads of 150 bp. Uniquely mapped rates were >60%.
Antibodies	H3K4me3 (C42D8) and H3K9me3 (D4W1U) from Cell Signaling Technology.
Peak calling parameters	HOMER v4.8 (Heinz et al. Mol. Cell 2010;38:576–589) was used to identify regions of the genome where more reads are present than random with default parameters for histone marks: 4.0 fold enrichment over input tag count; 0.0001 Poisson P-value threshold relative to input tag count; 4.0 fold enrichment over local tag count; 0.001 false discovery rate; 500bp peak size; 1000bp minimum distance between adjacent peaks. Differential peaks between tissue types were called using the findPeaks command with the control used as input as described in the Homer documentation.
Data quality	Quality control of the raw fastq files was performed using the software tool FastQC v0.10.1 and indicated high quality raw data for all samples. Average numbers of H3K4me3 and H3K9me3 peaks were 19,000 and 7,000, respectively.
Software	ChIPseq data were analyzed with the pipeline tool Omics Pipe (Fisch et al. Bioinformatics 2015;31:1724-1728) using the ChIPseq_HOMER pipeline running HOMER v4.8 (Heinz et al. Mol. Cell 2010;38:576–589). Code for downstream analyses and summarization of the Homer results files is available in Jupyter-Genomics (https://github.com/ucsd-ccbb/jupyter-genomics/blob/master/notebooks/chipSeq/Omics_Pipe_ChIPseq_GUI.ipynb).
